# A Longitudinal Study of Coping Strategies and Differences by Sex in Patients with Chronic Low Back Pain

**DOI:** 10.3390/jcm15020516

**Published:** 2026-01-08

**Authors:** Xavier Pericot-Mozo, Gloria Reig-Garcia, Afra Masià-Plana, Miquel Sitjar-Suñer, Carme Bertran-Noguer, Josefina Patiño-Maso, Rosa Suñer-Soler

**Affiliations:** 1 Department of Nursing, University of Girona, 17003 Girona, Spain; javier.pericot@udg.edu (X.P.-M.); gloria.reig@udg.edu (G.R.-G.); afra.masia@udg.edu (A.M.-P.); miquel.sitjar@udg.edu (M.S.-S.); carme.bertran@udg.edu (C.B.-N.); josefina.patino@udg.edu (J.P.-M.); 2 Health and Health Care Research Group, Department of Nursing, University of Girona, 17003 Girona, Spain; 3 Health Psychology Research Group, Department of Nursing, University of Girona, 17003 Girona, Spain

**Keywords:** low back pain, health-related quality of life, functional limitation, coping strategies, catastrophizing, resilience, nursing care

## Abstract

**Background/Objectives**: The most relevant psychological constructs for responding to stress in chronic lumbar pain in a positive way are active coping strategies, positive emotions, and resilience. The aim of this study was to study the coping strategies used by people affected by chronic low back pain and associated factors. **Methods**: We carried out a prospective longitudinal study involving people on a first visit at the Pain Unit of Josep Trueta University Hospital (Girona, Spain) presenting with chronic back pain, with a follow-up at three months. The Brief Pain Inventory (BPI), the Vanderbilt Pain Management Inventory (VPMI), and the Abridged Connor–Davidson Resilience Scale (CD-RISC) were used. The correlation of variables was analyzed, and a multiple linear regression model was used. **Results**: A total of 129 people with a mean age of 62.5 years participated (58.1% women). The mean severity of pain was moderate with mild improvement at the follow-up (6.42 to 6.17 points). The use of active coping strategies declined during the study (21.28 to 15.6 points), and a significant increase in passive strategies (23.6 to 30.21 points) and in catastrophizing (13.98 to 14.56 points) was observed. The total resilience scores were slightly better at baseline than at follow-up (27.50 to 26.67 points). The intensity of the back pain had a direct and significant relationship with passive strategies and an inverse relationship with active strategies and resilience. **Conclusions**: The coping strategies for dealing with chronic back pain observed in the study participants are not fully effective. The intensity of pain is significantly associated with the use of passive strategies and female sex.

## 1. Introduction

Chronic low back pain (CLBP) is the main health problem relating to diseases of the locomotor system and the main chronic disorder in the adult population. This musculoskeletal disorder, which has multifactorial causes [1,2,3], has a great impact on all dimensions of one’s quality of life, resulting in negative physical, psychical, social, family, occupational, and economic consequences [4,5,6]. CLBP has been identified as a prevalent long-lasting chronic disorder affecting between 12 and 30% of the global population [7,8] and increasing in prevalence with age [1,2,8].

Functional limitations are the main negative effect and are of moderate to high se-verity in most affected individuals, impacting their relationships, ability to work, and daily lives [9]. This limitation positively and significantly correlates with age, pain in-tensity, worse perceived health, the lack of personal autonomy, and mental disorders. These results are worse in women and older people [8]. Women, who have important anatomical and physiological differences compared with men (weaker pelvic floor muscles, lower estrogen levels and bone mineral density in pre- and post-menopausal stages), have more risk factors than men. Women continue to have a family burden (childhood stage), which is currently more accentuated at social and work levels (phys-ical burden) [1,10].

There is evidence that CLBP correlates strongly with mental health disorders, such as anxiety (concern, unrest, worry) [11], depression [12], and even generalized hyperal-gesia (also present in acute pain) [13], which correlate with a worse perception of pain and of health [14]. The most relevant psychological constructs in responding to the stressful situation caused by chronic back pain are active coping strategies, positive emotions [15,16], and promote resilience [17,18].

Different models and theories have evolved over the last four decades to explain the coping strategies of people with chronic pain. These have ranged from cognitive behavioral theories, such as Bandura’s self-efficacy theory (1977) and the stress model of Lazarus and Folkman (1984) [19,20], to more recent models such as biopsychosocial models, which grant more importance to the experience of pain taking into account personality, the acquisition of personal skills to adapt to it (resilience) [21], the acceptance of pain, and how to interact positively socially and with one’s environment.

These theoretical models are especially useful to understand how people interpret and cope with chronic pain [19,20]. Coping strategies are adaptive intrapsychic efforts that people adopt when faced with stressful events such as chronic pain. Each person will adopt a specific coping strategy depending on their own cognitive and behavioral evaluation of the stressor and the personal internal resources and external resources perceived as being available to them [22,23].

These efforts will determine how the pain experience is perceived and how the person adapts to limitations in daily life activities, which act as mediators of emotions [24]. There are several types of behavioral cognitive coping strategies, but generally they are considered to fall into two large groups. Active strategies are positively and significantly related with better adaptation, acceptance [6,24], protection, reduction, and regulation of the CLBP, better biopsychosocial tolerance of the pain, less functional limitation, better perception of health, and greater resilience, whereas passive strategies are positively and significantly related to worse adaptation, dependence, psychological deterioration, catastrophizing, isolation, social limitations and greater disability [15]. Cultural differences have a significant impact on both symptoms and behavior [25]. Regarding sex, women have more coping strategies for CLBP, despite a greater perception of pain [14].

Multidimensional and multidisciplinary behavioral cognitive rehabilitation therapies [26,27] are found to obtain significantly more effective and prolonged improvement in the pain symptoms with a moderate quality of evidence [28,29]. Perceived experience, expectations of efficacy, acceptance, and proactivity [30] are predictive psychological variables in the experience of pain [31] and are associated with better adaptation [6], maintenance, and recovery [32]. Pain unit nursing interventions through the use of a process management methodology are essential to achieve these objectives with quality, safety, efficacy, and healthcare efficiency [33].

The common practice in clinical practice has been to take a technical and pharmacological approach to the treatment of CLBP. When scientific research has studied coping strategies and resilience, it has tended to study the population as a whole without analyzing sex or gender differences. Given the scarcity of studies in this line, the present research has considered it essential to consider differences in coping strategies and resilience by sex.

## 2. Materials and Methods

### 2.1. Aims

The main aim was to study the coping strategies used by people affected by CLBP and the associated factors by sex. To this end, we describe the participants’ general characteristics, etiology, risk factors, and related clinical variables to determine pain intensity and to analyze coping strategies and resilience levels. Our hypothesis was that people who have a greater ability to cope or adapt have less perceived CLBP.

### 2.2. Design

A longitudinal, observational, and prospective design to study people with CLBP was undertaken at the Pain Unit of Josep Trueta University Hospital of Girona with a follow-up at three months.

### 2.3. Participants

The non-probabilistic sample was made up of all patients making a first visit to the Pain Unit between 1 December 2018 and 31 July 2019 with a diagnosis of CLBP as the main reason for the visit and who voluntarily agreed to participate in the study. The sample was non-probabilistic and consecutive, and participants were obtained from first visits made to the Pain Unit of the study center.

The inclusion criteria of the participants were to be aged 18 years or older, diagnosed with CLBP with or without radiculopathy, to be resident in the Girona Health Region, and to have provided their informed consent to participate in the study. The exclusion criteria were being younger than 18 years of age, not having been diagnosed with CLBP, and having cognitive impairments that impeded one’s ability to respond to the study questionnaires.

### 2.4. Data Collection

An ad hoc self-administered questionnaire was designed to record sociodemographic, clinical, and lifestyle variables, as well as details of personal daily life, family, occupation, and social aspects.

To study pain, the Spanish version of the Brief Pain Inventory (BPI) of Daut et al. [34,35] was used, which has been found to grant good reliability, with a Cronbach alpha > 0.70 for each of the dimensions. The BPI is a multidimensional instrument that evaluates pain characteristics, measuring different points in the perception of the intensity of pain, the localization of pain, the amount of relief provided by the treatment, and how the daily life activities of the participants are affected [36]. The questionnaire is made up of 15 items. All responses are provided on a numerical scale from 0 to 10, where 0 corresponds to not having pain, and 10 corresponds to the worst pain imaginable.

Coping strategies have been evaluated with the Spanish version of the Vanderbilt Pain Management Inventory (VPMI) of Brown Nicassio [37,38], which has good internal consistency, with a Cronbach’s alpha of 0.64 for active strategies (Cronbach’s alpha of 0.74 in the present sample) and 0.70 for passive ones (0.76 in the present sample). It is made up of 18 items [39]. Each response is evaluated through a Likert-type frequency scale with four options for each item, recodified with the following numerical values: 1 if the response was “almost never”, 2 for “sometimes”, 3 for “frequently”, and 4 for “almost always”, running from less to more coping strategies. The questionnaire evaluates the frequency with which people use specific active or passive coping strategies and four dimensions of coping: catastrophizing, seeking out social support, behavioral coping, and suppression. Higher scores indicate a greater use of strategies [40].

Resilience was evaluated with the Spanish version of the Abridged Connor–Davidson Resilience Scale (CD-RISC-10) [41,42], which has good internal consistency, with a Cronbach’s alpha of 0.91 [43] and 0.88 in the present sample. This scale is made up of ten items, and each response is evaluated using a five-option Likert-type scale, recodified with the following numerical values: 0 if the response was “never”, 1 for “rarely”, 2 for “sometimes”, 3 for “often”, and 4 for “almost always”. Higher scores indicate a good level of resilience and are associated with better mental health.

All these instruments are short, easy to complete, internally consistent, reliable, and validated, and they cover all the dimensions that we aimed to examine in this study.

### 2.5. Description of the Procedure and Data Collection

At the outset, the investigator orally provided all the information regarding the research project to people making a first visit to the Pain Unit. If they agreed to participate and met the inclusion criteria, they were provided the study information sheet and were asked to sign the informed consent form. The first phase of data collection was conducted by the investigator at the nursing clinic. All the questionnaires were self-administered, and clarification was provided if help was needed. The second phase of data collection was undertaken by the investigator at the same nursing clinic three months after the collection of the first data. Here again, the participants filled in the same questionnaires as before. The principal investigator verified that the questionnaires were correctly completed. All the study participants filled in the questionnaires both in the first and second phases.

### 2.6. Ethical Considerations

The research plan was approved by the IDIBGI Ethical Committee of our reference area. This study respects the ethical principles of research set out in Organic Law 15/1999 on the Protection of Personal Data, respecting the Code of Good Clinical Practice and always maintaining the confidentiality of patient data in accordance with Regulation (EU) 2016/679 of 27 April 2016 regarding the processing of personal data and the free movement of such data.

### 2.7. Statistical Analysis

The statistical study was performed using IBM SPSS 27 software. Continuous variables are described as the mean and standard deviation or the median and interquartile range. Categorical variables are described by the absolute frequency and their percentages. The chi-squared test and/or the Fisher test were used to study associations between categorical variables. Quantitative variables were compared using a non-parametric test, given that some coping variables did not follow a normal distribution. The Mann–Whitney U test was used to compare two independent samples, and the Wilcoxon signed-rank test was used to compare two related samples. In addition, a logistic regression model was performed to explain the relationship between the intensity of low back pain and the different coping strategies (active strategies, active–passive strategies, resilience) at the three-month follow-up of participants. In all the tests, significance was obtained as *p* < 0.05 with a 95% confidence interval.

## 3. Results

A total of 129 people, with a mean age of 62.5 years, were included (SD, 15.29). Ages were between 21 and 89 years, and 58.1% were women. In total, 41.1% of the participants were retired and 72.1% lived in a family. An amount of 41.9% of participants were not fully autonomous, requiring the help of others for the performance of basic daily life activities, and this figure was higher in women (45.3%).

The causes of the CLBP were mechanical; this cause is degenerative and was accompanied by radicular pain in the lower limbs (93.8%) in all age groups. This figure was higher in women, although statistical significance was not reached (92.6% vs. 94.7%, *p* = 0.719). Diagnoses were confirmed by the anesthesiologist of the Pain Unit. The most frequent surgical interventions performed on the participants prior to the study were lumbar discectomy (52.4%) and lumbar arthrodesis (47.6%); a total of 89.1% of these interventions followed pharmacological treatment. The main analgesic used was paracetamol (36.4%), followed by gabapentinoids (35.7%) and anticonvulsants (34.1%); the general consumption of analgesics was higher in women, although this was not significant (85.2% vs. 92%, *p* = 0.258).

Regarding psychological risk factors, 73.6% of the participants manifested having mental health disorders, such as anxiety (93.8%), depression (87.6%), and sleep disorders (58.9%), and these were significantly higher in women (63% vs. 81.3%, *p* = 0.026).

The potentially modifiable risk factors regarding observed lifestyle were sedentarism (91.1%), no leisure activities (60%), being overweight (33%), and obesity (37.5%), which were all higher in men but especially so in the case of obesity (BMI of 30.8 in men and 28.5 in women, *p* = 0.032). The occupational risks detected were maintaining a constant intense physical overload with bad posture (58.9%), which was higher in men (68.5% vs. 52%, *p* = 0.071), and occupational stress (31%), which was higher in women (35.2% vs. 28%, *p* = 0.442).

### 3.1. Intensity of the Perceived Pain in Daily Life Activity in People with CLBP at the Follow-Up

Slightly better scores were observed at the follow-up in pain intensity and in the extent to which daily life activities were affected. Specifically, statistically significant differences were seen in the maximum intensity of the pain on the 0–10 Numeric Rating Scale (7.76 vs. 7.47 points, *p* = 0.015), mean intensity of the pain (6.42 vs. 6.17 points, *p* = 0.055), impact on mood (7.91 vs. 7.27 points, *p* < 0.001), impact on individual work (6.82 vs. 6.50 points, *p* = 0.023), impact on relations with family and friends (5.85 vs. 5.50 points, *p* = 0.018), and impact on sleep (4.57 vs. 4.19 points, *p* = 0.026), and thus, it can be seen that women scored significantly worse for all items. On the other hand, men manifested a greater perception of pain relief (34.81% in men and 26.73% in women, *p* < 0.001).

### 3.2. Perceived Coping Strategies of People with CLBP at the Follow-Up

A reduction in the scores for active general strategies and an increase (which was similar in both sexes) in passive strategies were observed at the follow-up. A slight decrease in the scores for the specific behavioral strategies and an increase in the scores for the strategies of suppression, catastrophizing, and seeking out social support, without differences by sex (*p* > 0.05), was also found (Table 1).

Regarding the results obtained in responses to the VPMI questionnaire, and highlighting the highest percentages at the follow-up based on the strategies used, 63.6% of the participants responded that they almost always had catastrophic thoughts such as that they were fed up with the pain and that it was too much for them (63% vs. 64%, *p* = 0.225), and 51.2% indicated that they started thinking about how much and where it hurts (48.1% vs. 53.3%, *p* = 0.218), with higher scores in women. In the case of suppression strategies, 45.7% responded that they were almost always trying not to feel angry, depressed, and anxious (40.7% vs. 49.3%, *p* = 0.262), and 45% indicated that they almost never forgot the pain (38.9% vs. 49.3%, *p* = 0.182), with scores that were higher in women. Regarding behavioral strategies, 70.5% referred to almost always taking medicine to see whether the pain would go away (72.2% vs. 69.3%, *p* = 0.225), with scores that were higher in men, and 72.9% responded that they almost never did physical exercise (66.7% vs. 77.3%, *p* = 0.262), a figure which was higher in woman. In seeking out social support strategies, 53.5% almost always reduced their social activities (53.7% vs. 53.3%, *p* = 0.904), 91.5% almost never called a doctor or nurse (94.4% vs. 90.7%, *p* = 0.562), and 51.9% “prayed” for it not to hurt so much (63% vs. 44%, *p* = 0.125), with scores that were higher in men.

In comparing the means of the total scores for the coping strategies at baseline and follow-up, an increase in the passive scores, a decrease in the active strategies, and an increase in the scores for catastrophizing at the follow-up were all observed and significant (*p* < 0.001) (Table 2).

### 3.3. Perceived Resilience of People with CLBP at Follow-Up

A decrease in the scores for resilience was observed at follow-up, with men scoring better (*p* > 0.05) (Table 3).

Taking together the “often” and “almost always” responses of the CD-RISC-10 questionnaire at follow-up and highlighting the highest percentages, it was observed that 68.2% of the participants considered that they were able to cope with any situation (66.6% in men and 69.4% in women, *p* = 0.326), 65.1% that they recovered quickly from common illnesses (74.1% in men and 58.7% in women, *p* = 0.452), and 64.3% considered themselves to be strong in the face of life’s challenges (74.1% in men and 57.3% in women, *p* = 0.406), without significant differences between sexes.

When the means of the total scores for resilience at baseline and follow-up were compared, it was observed that the scores had declined significantly (27.50 vs. 26.67 points, *p* < 0.001, Wilcoxon signed-rank test).

### 3.4. Relationship of CLBP at Follow-Up with Age, Intensity of Maximum Pain, Coping Strategies, and Resilience

In participants who adopted more active coping strategies, more suppression strategies, and had greater resilience, these characteristics correlated positively with lower intensity of pain, fewer passive strategies, less catastrophizing, and less seeking out social support (*p* < 0.001).

### 3.5. Multiple Linear Regression Model

In the multiple linear regression model analyzing the intensity of the CLBP experienced by the participants at the three-month follow-up, an inverse relationship was detected between active coping strategies and resilience. In other words, the fewer the active strategies and the lower the resilience, the greater the perceived pain intensity, and there was a direct relationship with passive strategies in that the greater the number of passive strategies, the greater the intensity of the CLBP.

Table 4 shows the factors associated with the intensity of back pain at the three-month follow-up. This intensity is associated with sex in that women had significantly higher scores (*p* = 0.004) and a greater use of passive strategies. In other words, the more passive strategies were employed, the greater the perception of pain (*p* < 0.001).

## 4. Discussion

The present study investigated the coping strategies of 129 people affected by CLBP over a three-month period and the associated factors by sex.

Regarding the general characteristics of the people studied, our sample had a mean age of 62.5 years, which is similar to that of Lee et al. [44] but higher than other studies [45]. A total of 58.1% of the participants were women, which was a similar proportion to the 58.4% of Tyack et al. [46] and the 57.7% of Jegan et al. [47]. According to the systematic review of Meucci et al. [10], differences in proportions by sex could be related to the greater tasks taken on by women in the family setting, biological characteristics, and the hormonal processes of women [1,48].

CLBP has a great impact on functional limitations. The clinical guides and a systematic review [49] point to moderate evidence that the performance of regular mild exercise is the most effective conservative treatment with the most significant clinical differences in reducing the perception of pain and functional limitation in undertaking daily life activities [9,49,50,51]. In the present study, looking at modifiable risk factors, it was found that most participants did not perform any leisure activity in their free time, and those who did had difficulties performing them due to the persistent functional limitation provoked by the intensity of the back pain. Men manifested greater concern in this regard. Given that leisure and distraction are considered to be coping strategies to reduce perceived pain [14], we note that there was a clear lack of such strategies in the participants in our study. Hong and Shin also observe a high rate of sedentarism (computer use time) in both sexes [52], with men being significantly more overweight than women; the findings of Quentin et al. [45] and Ruiz et al. [50] were similar to those in the present samples, but Ünal et al. [53] observed that these findings occurred to a lesser degree in their results.

CLBP also has a great impact on mental health, with a strong positive correlation between the severity of pain, depression, anxiety, sleep disorders [12,18,52], negative emotions [12]. In the present study, it is important to highlight that seven out of ten participants indicated that they had mental health disorders; this rate was significantly higher in women, in line with the findings of other authors [54], where loss of control and catastrophism were observed. These are comorbidity factors [55] and important factors of bidirectional and non-causal prediction [12].

CLBP also has a major impact on people’s employment situation. Although in the present research, four out of ten participants were retired, those who were working had great difficulty in performing their usual professional activity with normality, undergoing occupational stress, absences from work, and long and frequent periods of temporary incapacity, with percentages that were higher among women [1,56].

### 4.1. The Intensity of the Back Pain

In the present study, the perception of CLBP found among the participants at follow-up was of a moderately high intensity, which was similar to the findings of Zavarize and Wechsler [14], although there was greater severity and affectation in all basic aspects of their lives in our study.

Regarding pain relief associated with pharmacological treatment of the participants in the study, a certain improvement was observed at follow-up (23.22% vs. 30.12%), and men continued experiencing more pain relief. The most effective therapeutic intervention observed was the use of epidural steroid injections (showing Level I evidence) performed during the period of the study, with an improvement of two points in the maximum pain intensity and in the mean pain intensity, which was similar to the findings of other studies that used local anesthetics and steroids [57]. Rehabilitation treatments and educational interventions have been found to reduce perceived pain by more than five points on the 0–10 Numeric Rating Scale (from 7.17 to 2.78 points) [31] and to improve functional limitation [58].

### 4.2. Coping Strategies

In the present investigation, the participants initially adopted adaptive skills with an active coping style, and their efforts were focused on the problem, reducing or eliminating the stressor. This has also been observed by Garcia et al. [56] (86.86% used active strategies, and 13.14% used passive strategies). However, at the follow-up, the contrary situation is found, with the participants manifesting a lack of adaptive skills and a passive coping style, ceding the management and treatment of the CLBP to other people while their own efforts were centered on emotional reactions, as was also found by Ruiz et al. [50]. In this context, the continued adoption of these maladaptive strategies predicts worse cognitive and behavioral coping and worse adaptation to the pain experience and is related to a greater degree of stress, worse psychological wellbeing, greater perceived pain [15,24], greater functional limitation, worse perceived health, and lower likelihood to seek out perceived social support.

Maintaining behavioral strategies that are influenced by negative emotions may lead to the presentation of a greater intensity of pain, more functional limitation, worse perceived health, and worse adaptation and recovery [15]. During the follow-up, the participants had difficulty adopting positive behavioral efforts with attempts to reduce or tolerate the stressful situation, which, according to Ho et al., can improve through education and cognitive behavioral therapy [59]. Constant hypervigilance of pain behavior, lack of distraction/leisure, and not being able to undertake physical activity due to pain while maintaining adequate pharmacological treatment were observed in the present study. The avoidance of mobilization due to fear of pain has been found to provoke progressive physical deterioration of the musculoskeletal system [60].

Action inhibition is a normal attitude for coping with stressful and distressing experiences that people with CLBP perform as an efficient short-term coping strategy. It was observed that participants maintained continued efforts of suppression, which are not beneficial nor adaptive in the modulation of the pain experience, because this blocks the expression of annoying feelings, as well as positive thoughts and emotions, and is associated positively and significantly with the severity of pain, more muscular contractions, and depression, as has been found in other studies [61].

Furthermore, the participants expressed having difficulty in managing to expel negative thoughts and emotions from their field of consciousness, as in Pegram et al. [16]. Maintaining efforts to suppress anger leads to greater muscular tension and greater severity of pain [62]. It should also be highlighted that maintaining an intentionally evasive coping style in the workplace and awaiting a judicial process related to disability to ob-tain economic compensation are related to a worse perception of CLBP and of health [63].

Several studies have shown that catastrophizing is an important psychosocial risk factor that is positively and significantly related to the appearance, maintenance, and chronification of the intensity of pain [64,65] and more muscular contractions. In our study, a statistically significant increase in the more frequent use of catastrophizing thoughts and behavior was observed at the follow-up, which is in line with other studies [50,66]. The participants were not capable of using their efforts to face up to the stressful situation, and they tended to have exaggeratedly intense negative perceptions with constant exaggerated thoughts and to worry excessively in a way that was threatening, unsettling, and out of control regarding their pain experience. On the other hand, in young people without chronic pain, catastrophizing is the least used strategy. Carriere et al. [67] add that expectations and negative experiences have an impact on catastrophic thoughts.

In the present study, a slight increase was observed at follow-up in the use of strategies of seeking out social support. The participants in the study did not have effective social skills to face the stressor. The family was the main source of physical and psychological help and the main outlet for the expression of emotions. The participants’ efforts were not aimed at facing up to the problem (the lack of help, isolation, reduction in activity), but rather, participants depended on others to help them, which is a similar finding to that of other studies [16,50].

Regarding sex, women more often used passive general strategies (catastrophizing and seeking social support) and more behavioral strategies to manage the CLBP. Similar results were found by Zavarize and Wechsler [14]. Moreover, Cabak et al. [30] and Baker et al. [68] found that older people had more active strategies than did younger age groups and that this difference was significant. On the other hand, men more often used active general strategies and suppression. However, a larger sample will be necessary to confirm these results.

### 4.3. Resilience

Very few studies have associated resilience with CLBP. In our study, the participants did not consider that they had great qualities and/or positive abilities in maintaining adaptive skills and recovering adequately from stressful situations. Total scores for resilience were found to be mid-range both at baseline and follow-up, similar to the scores found by Jegan et al. [47].

Regarding sex, few studies relate sex with resilience and CLBP, except that of Zavarize and Wechsler [14], who obtained similar results to those of the present study. It has been pointed out that resilience is significantly associated with personality char-acteristics, more coping, more self-affirmation, more mental self-control, more search-ing for information about the disease, less catastrophizing, less fear or avoidance of movements [18], a slower pace of biological aging, and better pain outcomes [21]. O’Keeffe et al. [58] and Bartley et al. [17] highlight the need to work on educational in-tervention programs to improve resilience skills and to learn to cope better with diseas-es in general [69,70].

Nursing professionals in pain units aim to promote self-treatment skills, autonomy, and empowerment among patients and family members to achieve optimum coping with chronic back pain [24]. We help to design and direct prevention strategies to avoid worsening and the derived complications through specific functions such as the programming of visits by priority, case management, the coordination of different care services [9], health education [71], emotional support, and especially, using telephone consultation to reduce the number of medical consultations. Furthermore, we help to avoid displacements, following up on the pain and its treatment (secondary effects) and establishing care liaison with both the patient and primary care [33]. All of this translates into better care quality with better results regarding perceived health and less healthcare expenditure.

While the results of the present study confirm the hypothesis that people who presented better adaptation (*coping strategies*) and greater resilience (*that can modulate strategies*) to CLBP would present less perception of pain and would use more active strategies in dealing with the CLBP, which is in line with other studies [72,73], we did not find differences by sex in the style of coping in our sample, which is also in line with other authors [74], but it differs from other studies [75].

Having said that, it should be noted that this is one of only a few studies to analyze the relationship between the characteristics of patients with CLBP, the etiology of the pain, risk factors, clinical variables, and pain intensity with psychological variables and to examine the association of these variables with coping strategies and resilience. As such, it can be considered a new line of investigation that aims to increase our understanding of the process of adaptation to the experience of chronic pain and how it affects the daily life of people with CLBP.

### 4.4. Limitations

Two of the main limitations of this study are the observational design, which does not allow causal relationships to be drawn, and the short period of follow-up. Moreover, the exact onset of chronic low back pain was not evaluated on inclusion since the patients were imprecise in reporting how long they had been experiencing pain before being studied at the hospital. A further limitation is the difficulty in comparing some results with other studies due to the use of different measurement instruments, as, for example, few authors have used the Vanderbilt questionnaire regarding CLBP, and fewer still have looked at the association of the constructs of coping strategies and resilience with CLBP.

## 5. Conclusions

People with chronic low back pain were found to use a catastrophizing coping strategy most often, and they were found to use behavioral coping strategies or the strategies of suppression or seeking out social support the least. In other words, their capacity to cope with pain and be resilient was not as effective as it could be. The intensity of pain is found to be significantly associated with the use of passive strategies and female sex. However, both sexes obtain similar scores in active and passive coping strategies and in resilience.

Physical, cognitive, and behavioral rehabilitation, as well as multidisciplinary interventions, should be promoted to improve modifiable lifestyle, functional, occupational, mental health, and social risk factors. Learning to use more coping strategies will permit people to adapt better to living with CLBP.

## Figures and Tables

**Table 1:** Coping strategies at baseline and follow-up for the overall sample and by sex.

Vanderbilt Questionnaire	Baseline				During Follow-Up			
	Sample Mean (SD) Median [Range]	Men Mean (SD) Median [Range]	Women Mean (SD) Median [Range]	*p*	Sample Mean (SD) Median [Range]	Men Mean (SD) Median [Range]	Women Mean (SD) Median [Range]	*p*
**General strategies**								
Active	21.28 (8.34) 20 [14–28]	21.48 (7.77) 21 [15.75–28]	21.13 (8.7) 20 [14–28]	0.589	15.6 (4.38) 15 [13–18]	15.79 (4.37) 16 [13–18.75]	15.47 (4.42) 15 [12–18]	0.642
Passive	23.6 (8.98) 23 [16–30.5]	23.20 (8.39) 23 [15–29.25]	23.88 (9.42) 23 [16–32]	0.713	30.21 (5.95) 30 [25–35]	29.63 (5.47) 29 [25.25–34]	30.61 (6.27) 31 [25–35]	0.412
**Specific active strategies**								
Behavioral	11.93 (2.97) 12 [9–14]	11.81 (3.35) 12 [9–14.25]	12.01 (2.67) 12 [10–14]	0.699	11.74 (2.8) 12 [9–14]	11.54 (2.97) 12 [9–14]	11.88 (2.7) 11 [10–14]	0.634
Suppression	10.35 (2.73) 10 [8–13]	10.70 (2.7) 10 [8.75–13]	10.09 (2.74) 10 [8–13]	0.214	10.42 (2.54) 10 [8–12]	10.65 (2.48) 11 [8–12]	10.23 (2.59) 10 [8–12]	0.360
**Specific passive strategies**								
Catastrophism	13.98 (3.96) 14 [11–17.5]	13.81 (3.86) 14 [11–17]	14.09 (4.06) 14 [11–18]	0.768	14.59 (3.34) 14 [12–17]	14.54 (3.19) 14 [12–16.75]	14.57 (3.46) 15 [12–18]	0.976
Seeking out social support	8.88 (2.80) 9 [7–11]	8.35 (2.65) 8 [6–10.25]	9.25 (2.86) 9 [7–11]	0.070	9.07 (2.66) 9 [7–11]	8.6 (2.53) 8 [6–10.25]	9.40 (2.71) 9 [7–11]	0.074

*Note.* Mann–Whitney U test. The results are expressed with means, standard deviation (SD), and median and interquartile range [IQR].

**Table 2:** Comparison of the median of the overall scores for the coping strategies at baseline and during follow-up.

Coping Strategies	Baseline	Follow-Up	*p*
	Median [IQR]	Median [IQR]	
**Active**	20 [14–28]	15 [13–18]	<0.001
**Passive**	23 [16–30.5]	30 [25–35]	<0.001
**Behavioral**	12 [9–14]	12 [9–14]	0.017
**Suppression**	10 [8–13]	10 [8–12]	0.862
**Catastrophism**	14 [11–17.5]	14 [12–17]	<0.001
**Seeking out social support**	9 [7–11]	9 [7–11]	0.150

*Note.* Wilcoxon signed-rank test. The results are expressed with median and interquartile range [IQR].

**Table 3:** Total score for resilience at baseline and follow-up for the overall sample and by sex.

CD-RISC-10 Questionnaire	Baseline				During Follow-Up			
	Sample	Men	Women	*p*	Sample	Men	Women	*p*
	Mean (SD) Median [Range]	Mean (SD) Median [Range]	Mean (SD) Median [Range]		Mean (SD) Median [Range]	Mean (SD) Median [Range]	Mean (SD) Median [Range]	
**Score**	27.50 (8.75) 29 [22–35]	28.78 (8.17) 30.5 [24–36]	26.59 (9.09) 26 [21–35]	0.167	26.67 (8.79) 28 [21–33]	28.11 (8.17) 29 [23–34.25]	25.63 (9.13) 26 [19–33]	0.126

*Note.* Mann–Whitney U test. The results are expressed with means, standard deviation (SD), and median and interquartile range [IQR].

**Table 4:** The linear regression model relationship between the intensity of the pain and active and passive strategies and resilience.

	Unstandardized Coefficient		Standardized Coefficient	t	*p*	95% Confidence Interval for B	
	B	ES	β			Lower Limit	Upper Limit
(Constant)	0.219	1.664		0.132	0.896	−3.075	3.513
**Age**	0.018	0.012	0.127	1.548	0.124	−0.005	0.042
**Sex**	1.082	0.365	0.240	2.967	0.004	0.360	1.804
**Active strategies**	−0.014	0.047	−0.028	−0.301	0.764	−0.108	−0.079
**Passive strategies**	0.122	0.031	0.326	3.881	<0.001	0.060	0.184
**Resilience**	−0.015	0.023	−0.060	−0.648	0.518	−0.061	0.031

Abbreviations: B, unstandardized coefficient beta; ES, standard deviation error; β, standardized beta coefficient; *p* < 0.05; CI 95%, confidence interval of 95%; R^2^, 0.23; adjusted R^2^, 0.198.

## Data Availability

For ethical reasons, the full data presented in this study are only available upon reasonable request from the corresponding author.
